# Hydrate-based heavy metal separation from aqueous solution

**DOI:** 10.1038/srep21389

**Published:** 2016-02-18

**Authors:** Yongchen Song, Hongsheng Dong, Lei Yang, Mingjun Yang, Yanghui Li, Zheng Ling, Jiafei Zhao

**Affiliations:** 1 Key Laboratory of Ocean Energy Utilization and Energy Conservation of Ministry of Education, School of Energy and Power Engineering, Dalian University of Technology, Dalian 116024, People’s Republic of China

## Abstract

A novel hydrate-based method is proposed for separating heavy metal ions from aqueous solution. We report the first batch of experiments and removal characteristics in this paper, the effectiveness and feasibility of which are verified by Raman spectroscopy analysis and cross-experiment. 88.01–90.82% of removal efficiencies for Cr^3+^, Cu^2+^, Ni^2+^, and Zn^2+^ were obtained. Further study showed that higher R141b–effluent volume ratio contributed to higher enrichment factor and yield of dissociated water, while lower R141b–effluent volume ratio resulted in higher removal efficiency. This study provides insights into low-energy, intensive treatment of wastewater.

Hydrates are solid crystalline structures—comprising water (host molecules) and small molecules (guest molecules) such as CO_2_, N_2_, CH_4_, H_2_, and H_2_S—that are formed under conditions of low temperature and relatively high pressure. Guest molecules are enclosed within water cavities consisting of hydrogen-bonded water molecules^1^,^2^. There has been much interest in the applications of hydrates, and those containing natural gas guest molecules have received attention as a potential new energy source. Innovative technologies have been researched and developed on the basis of the physical and chemical properties of hydrates. Mohammadi *et al*. achieved carbon dioxide capture from a mixture of different gaseous compounds by analyzing hydrate phase equilibrium data and the conditions for hydrate formation and dissociation^3^,^4^,^5^,^6^,^7^,^8^. Tumba *et al*. conducted separation experiments of close-boiling point compounds according to the varying conditions under which each component forms hydrates^9^,^10^,^11^. In addition, refrigerant hydrates have high cold storage capacities and efficiencies, which led Hashemi *et al*. to investigate the conditions required for the formation and dissociation of refrigerant hydrates for applications in cool storage, refrigeration, and air conditioning systems^12^,^13^,^14^,^15^. Moreover, Strydom *et al*. studied the hydrate dissociation conditions of the refrigerant + sucrose in aqueous solution for use in the sugar milling processes as a means of increasing the solid content in aqueous carbohydrate systems^16^.

It is worth noting that the nature of separating heavy metals from aqueous solution by physical methods, especially the treatment of electroplating effluent, is also a physical separation process in which contaminants are removed from wastewater^17^. Electroplating effluent usually contains heavy metals such as copper, nickel, zinc, and chromium^18^,^19^,^20^,^21^, which are nonbiodegradable and bioaccumulative. These heavy metals are known to be toxic or carcinogenic^22^ and should be reduced to permissible levels prior to discharge to the environment. Various techniques have been employed for the treatment of heavy metals, including precipitation, electrochemistry, adsorption, ion exchange, and membrane filtration^19^,^21^,^23^. The precipitation method is based on chemical coagulation by adding certain chemical substances, followed by separate precipitation from the effluent^22^. Although it has shown high removal efficiency in treating wastewater containing heavy metals, the chemical coagulation process may induce secondary pollution due to the addition of chemical substances^24^ and the generation of hazardous sludge^25^. The electrochemical method requires the constant sacrifice of electrode material. Its drawbacks also include the formation of sludge and a passivation layer on electrodes^26^ in addition to high operational cost associated with energy consumption^21^. For adsorption, the recovery of adsorbent and the recycling of heavy metals are far more complicated. Although ion exchange has advantages over the above methods, suitable ion exchange resins are not available for all heavy metals, and the capital and operational costs remain high^27^. In terms of membrane filtration, the selection of an appropriate membrane involves factors such as the characteristics of the effluents, the properties and concentrations of materials present in the wastewater, pH, and temperature^28^; in addition, this approach has high operating and maintenance costs^29^.

Consequently, there is a growing need for alternative methods of treating effluent containing heavy metals, for which hydrate-based separation appears promising. Correlational research into separation and purification using a hydrate process has attracted scientific interest. As early as 1942, Parker proposed a method to produce potable water from seawater by hydrate formation^30^, which has recently received considerable attention. Hesse and Harrison observed a marked decrease in interstitial water chlorinities in deep-water sedimentary sections containing hydrate, and noted that hydrate excludes the salt ions from the crystal structure^31^, which provides the theoretical foundation for separating mixtures in a hydrate-based method. For separation of inorganic mixtures, Knox *et al*. proposed a process for the desalination of seawater to produce potable water and established a pilot plant to study the process^32^. Moreover, Bulot *et al*. proposed a process for forming purified solute from an aqueous mixture of water and solute^33^. Ngema *et al*. provided accurate phase equilibrium data for hydrate formation in saline solutions derived from experimental measurements and thermodynamic models. This data could be used to design wastewater treatment and desalination processes using hydrate technology^34^,^35^. For separation of organic mixtures, Huang *et al*. studied the concentrations of apple, orange, and potato juices using methyl bromide, trichlorofluoromethane, and 1,1-difluoroethane, and reported that their method removed 80% of the water content^36^. Bradshaw *et al*. assessed that hydrate desalination is more efficient in terms of water throughput and recovery when compared to reverse osmosis^37^. All of these studies indicate that hydrate-based methods can be applied to mixture separation.

Therefore, based on the above theory and previous achievements, a hydrate-based method is proposed for separation of heavy metals from aqueous solution. The removal effectiveness of this method with different R141b–effluent volume ratios was demonstrated by Raman spectroscopy and cross-check; the effect of a washing operation on the removal of heavy metal ions was investigated; the effect of R141b–effluent volume ratio on removal characteristics is discussed.

Aqueous solution was synthesized to simulate electroplating effluent in a hydrate-based experiment, using chromium chloride hexahydrate^38^, nickel sulfate hexahydrate^29^, zinc vitriol^26^, and copper sulfate pentahydrate^39^. Under atmospheric pressure at temperatures lower than 8.4 °C, hydrochlorofluorocarbon (HCFC) R141b (CH_3_-CCl_2_F) is known to form a structure-II hydrate consisting of a central organic molecule surrounded by 17 water molecules^40^; it was selected as the hydrate former^41^,^42^,^43^ in the present study because of its immiscibility with water, non-toxicity, and thermodynamic stability.

## Methods

**Figure 1:**
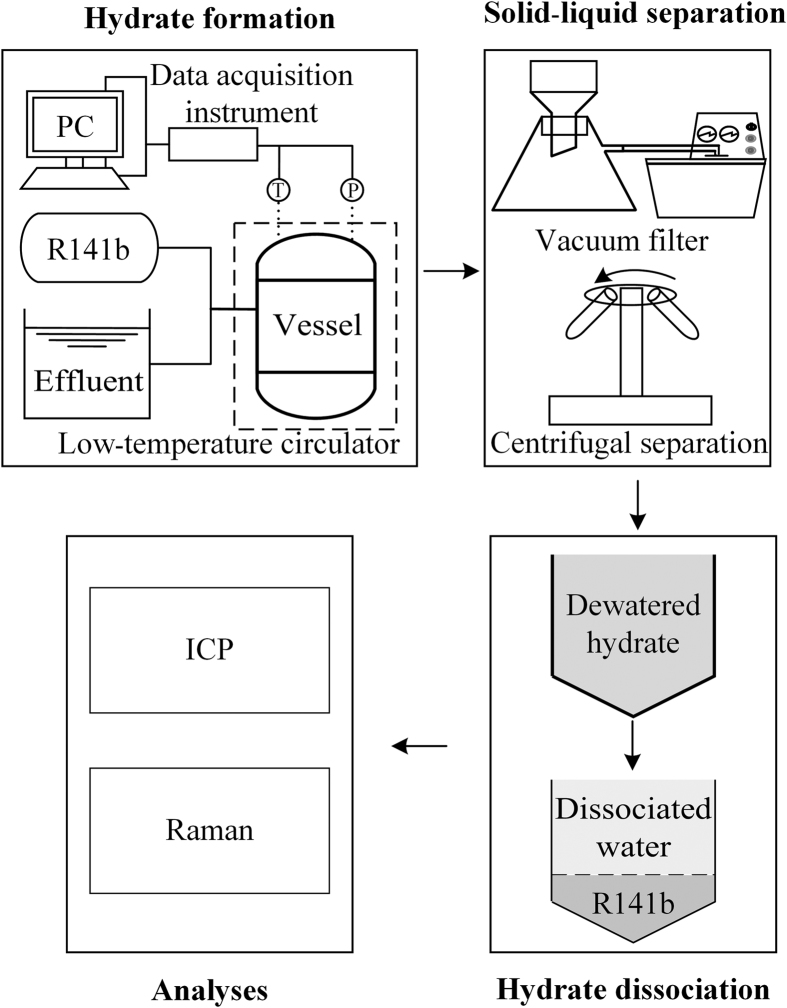
Experimental flow diagram of heavy metal separation from aqueous solution.

The experimental flow diagram is illustrated in Fig. 1. A stainless steel reactor with 100 mL internal volume, a thermocouple and pressure sensor was designed to carry out the hydrate formation experiment. During the experiment, the reactor was submerged in a low-temperature ethylene glycol circulator with precision of 0.01 °C to control the temperature^44^. The system was monitored via a data acquisition instrument. The liquid circulation system was turned on first to circulate the liquid and achieve a steady experimental temperature^8^. When the conditions inside the reactor reached atmospheric pressure and 4 °C^12^, ISCO pumps were used to inject the simulated electroplating effluent and hydrate former into the reactor in appropriate proportions. The operating temperature and pressure were held constant during the hydrate formation process^8^,^45^. The reactor was shaken every hour to enable hydrate conversion. During the experiment, a temperature spike was observed during hydrate formation, and then the temperature restored to the experimental temperature. To ensure that hydrate formation was complete, the following experiment was carried out once no temperature change had been observed for more than 480 minutes. After the hydrate was fully formed, as judged by the reaction time and temperature change^46^,^47^,^48^, the hydrate slurry containing hydrate and residual effluent first underwent vacuum filtration, the volume of residual effluent was measured and the hydrate was washed using deionized water spray (one-tenth initial effluent volume) at the selected operating temperature^49^,^50^. Then, centrifugal separation at 3000 r/min was conducted to further remove interstitial water. Each of the above procedures was conducted in a refrigeration chamber at a temperature of 3–5 °C. Next, the dewatered hydrate was transferred into a decomposer, where it decomposed into R141b and water at room temperature and ambient pressure^45^. Finally, R141b was separated from the mixture of R141b and water, based on its immiscibility with water, and could then be reused. In addition, each heavy metal ion concentration (Ni^2+^, Cr^3+^, Cu^2+^ and Zn^2+^) was measured by an inductively coupled plasma optical spectrometer. The liquid phase R141b, R141b hydrates formed in deionized water, and the R141b hydrates formed in electroplating effluent were washed liberally with water to remove ions adhered to the surface of the hydrate, and were then characterized via Raman spectroscopy to ascertain the removal mechanism.

Removal efficiency (Re) was calculated as follows^20^,^25^,^51^,^52^:
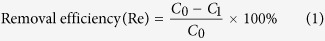
where C_0_ is the concentration of each heavy metal ion in electroplating effluent, and C_1_ is that in the dissociated water; C_1_ includes two parts: C_11_ is the concentration of each heavy metal ion in the dissociated water without the washing process, and C_12_ is that following the washing process. To characterize the residual effluent, the enrichment factor (Ef) was calculated as follows^20^:
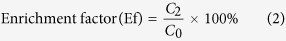
where C_2_ is the concentration of each heavy ion in the residual effluent. Additionally, the yield of dissociated water is calculated as follows:
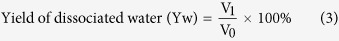
where V_0_ is the initial volume of simulated electroplating effluent, and V_1_ is the volume of dissociated water from hydrate dissociation.

In addition, tests were carried out in duplicate to ensure reproducibility of results. Each experiment was conducted four times. The specifications and sources of the experimental reagents and instruments are presented in Tables 1 and 2, respectively.

## Results and Discussion

**Figure 2:**
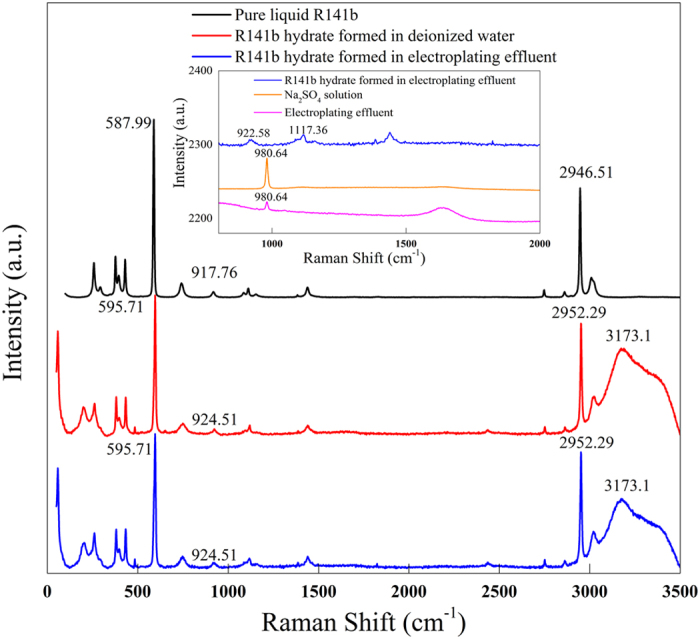
Raman spectra of pure liquid R141b, R141b hydrate formed in deionized water, and of simulated electroplating effluent.

During the heavy metal separation process, hydrate was formed under atmospheric pressure and 4 °C. First, extracted hydrate samples were characterized by Raman spectroscopy. A comparison of the R141b hydrate Raman spectra with that of pure R141b is shown in Fig. 2. In the mid-infrared region, the R141b spectrum is dominated by C–Cl and C–F stretch modes. The Raman spectroscopy results demonstrate that the characteristic peaks associated to C–Cl, C–F symmetric stretch have been shifted approximately 7 cm^−1^ higher than those of pure liquid R141b to R141b hydrate; this is attributed to interactions between the guest and the cage walls, and to the confining effects of the water cage, which lead to higher vibrational frequencies for bonded modes. Meanwhile, the CH_3_ symmetric stretch shifted from 2946.51 cm^−1^ in liquid R141b to 2952.29 cm^−1^ in R141 hydrate. An O–H stretch (3173.1 cm^−1^) is also observed only in R141b hydrate Raman spectroscopy. It is worth noting that no peak-shift is observed between the R141b hydrates formed in deionized water and electroplating effluent for C–Cl, C–F, CH_3_, and O–H bonds. This indicates that the metal ions in the water did not affect Raman peak position and the hydrate structure. From Fig. 2, the Raman peak associated to 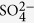 at 980.64 cm^−1^, which is confirmed by sodium sulfate solution which is only found in the electroplating effluent; in contrast, in R141b hydrate, there is no trace of 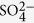. This implies that 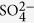 remained in the electroplating effluent rather than being encapsulated into the hydrate structure. Since the ionic interactions between 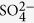 and metal ions are much stronger than the host–guest van der Waals forces in hydrates, heavy metal ions should also remain in the effluent together with the 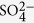 ions. This result was cross-checked by analyzing the concentrations of heavy metal ions in a sample of dissociated R141 hydrate that had been thoroughly washed with deionized water. The heavy metal ion in the dissociated water declined from about 140 mg/L to less than 0.4 mg/L after hydrate-based treatment, as shown in Table 3, demonstrating the exclusion of heavy metal ions from the hydrate structure.

**Figure 3:**
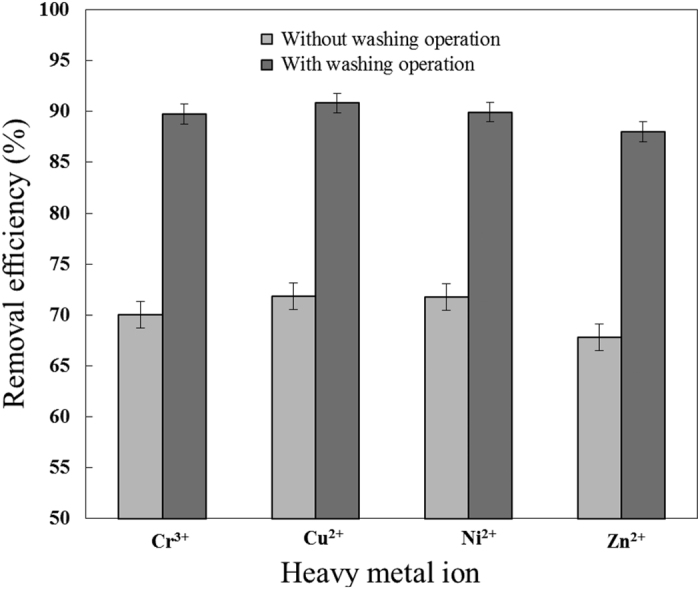
Removal efficiency at R141b–effluent volume ratio of 1:6.

The experimental conditions used for heavy metal separation are shown in Table 3. At an R141b–effluent volume ratio of 1: 6, the initial Cr^3+^ concentration in S2 was reduced from 96.70 mg/L to 28.99 mg/L by hydrate-based separation without washing operation, equivalent to 70.02% removal efficiency of Cr^3+^. Similarly, the removal efficiencies are approximately 71.87%, 71.79%, and 67.82% for Cu^2+^, Ni^2+^, and Zn^2+^, respectively. During the hydrate formation process, the residual effluent becomes concentrated in heavy metals because they are excluded from the hydrate cages. However, the high-concentration residual effluent is partially trapped in the porous structure of the hydrate and adhered to the hydrate surface. Therefore, relatively high concentrations of heavy metal ions remain in the dissociated water, resulting in low removal efficiency. To further remove heavy mental ions, a washing operation was performed, resulting in substantially increased removal efficiencies for Cr^3+^, Cu^2+^, Ni^2+^, and Zn^2+^ (range 88.01–90.82%; approximately 19% higher than separation without washing. See Fig. 3). Additionally, the enrichment factor of each heavy ion, which characterizes the difficulty of further treatment of residual effluent, reached approximately 1.3. The yield of dissociated water was 61.67%. These results indicate that the hydrate-based method is capable of removing heavy metal ions from electroplating effluent, and that the washing operation improved removal efficiencies.

**Figure 4:**
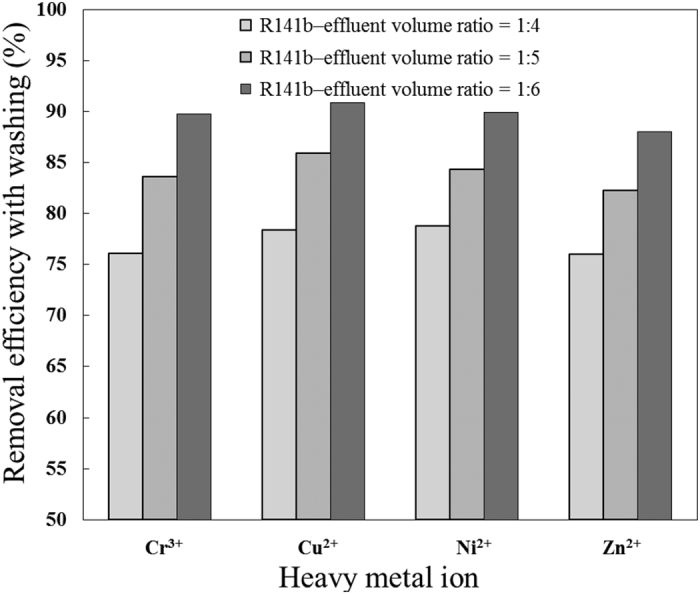
Effect of R141b–effluent volume ratio on removal efficiency with washing.

**Figure 5:**
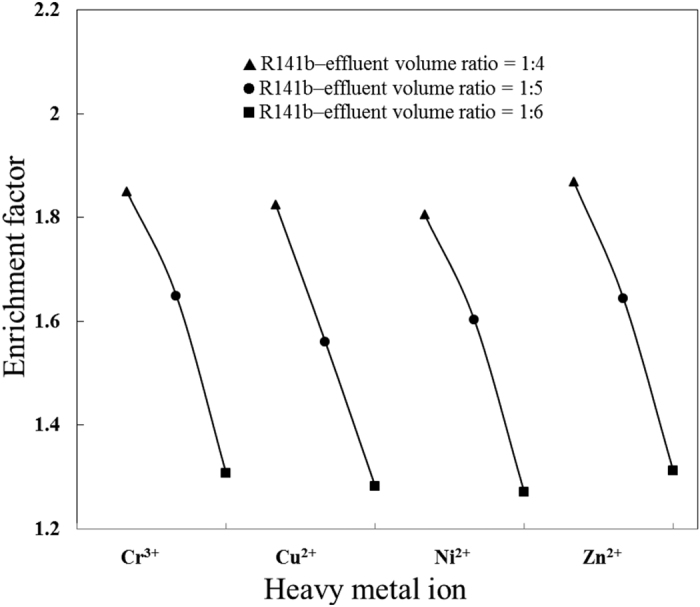
Effect of R141b–effluent volume ratio on enrichment factor.

S2, S3, and S4 were conducted to explore the effect of R141b–effluent volume ratios on the removal of heavy metal ions. As illustrated in Figs 4 and 5, by changing the R141b–effluent volume ratio from 1: 4 to 1: 6, removal efficiency increased while enrichment factor decreased. Noting that the removal efficiency and enrichment factor were approximately the same for all four heavy metal ions, regardless of different ionic radius and charges, these results were consistent with those reported by Cha^52^, in which high-salinity produced water including Na^+^, Mg^2+^, K^+^ and Ca^2+^ was desalinized by a gas hydrate-based process using cyclopentane and cyclohexane as hydrate formers. Removal efficiency is essentially dependent on the concentrations of heavy metal ions in the dissociated water (C_1_), whereas the enrichment factor relies on the concentrations of heavy metal ions in the residual effluent (C_2_). The R141b–effluent volume ratio determines the percentage of water conversion into hydrate. Theoretically, all the water would be converted to hydrate at an R141b–effluent volume ratio of 1:3.21. By gradually decreasing the R141b–effluent volume ratio, water consumption declines, resulting in lower concentration of heavy metal ions in the residual effluent (C_2_). Thus, fewer heavy metal ions are trapped between or adsorbed onto the surface of the hydrate crystallites. After the hydrate dissociated under ambient pressure and temperature, fewer heavy metal ions were present in the dissociated water (lower C_1_). In summary, higher R141b–effluent volume ratio contributed to higher enrichment factor and yield of dissociated water, but lower removal efficiency. Remediation could involve secondary treatment of dissociated water. On the other hand, lower R141b–effluent volume ratio results in higher removal efficiency but lower yield of dissociated water. This could be improved by subjecting the residual effluent to a second round of hydrate formation.

## Conclusions

This study proposes a hydrate-based method for separation of heavy metals from aqueous solution, the effectiveness and feasibility of which are verified by Raman spectroscopy analysis and cross-experiment. Raman spectroscopy analysis showed that the R141b hydrate peak is shifted approximately 7 cm^−1^ higher than that of the liquid R141b peak, whereas heavy metals did not affect the R141b Raman peak position and hydrate structure, indicating that the heavy metal in aqueous solution did not participate in the formation of hydrate. A washing operation increased the removal efficiencies for Cr^3+^, Cu^2+^, Ni^2+^, and Zn^2+^ by approximately 19% (from 67.82–71.87%). Further research showed that higher R141b–effluent volume ratio contributed to higher enrichment factor and yield of dissociated water, while lower R141b–effluent volume ratio resulted in higher removal efficiency. Despite the advantages of hydrate-based methods for separation of heavy metals, many challenges remain. Further studies are required on the selection of appropriate hydrate former and promoter, and solid–liquid separator to improve efficiency. It is our hope that the hydrate-based process for heavy metal separation proposed in this study might be effective for wastewater treatment.

## Additional Information

**How to cite this article**: Song, Y. *et al*. Hydrate-based heavy metal separation from aqueous solution. *Sci. Rep.* **6**, 21389; doi: 10.1038/srep21389 (2016).

## Figures and Tables

**Table 1:** Specifications and suppliers of reagents.

Material	Chemical formula	Purity	Supplier
Chromium chloride hexahydrate	CrCl_3_•6H_2_O	99.0%	Xilong Chemical Industry Incorporated Co., Ltd., Guangdong Province, P.R.C.
Nickel sulfate hexahydrate	NiSO_4_•6H_2_O	98.5%	Damao Chemical reagent Factory, Tianjin City, P.R.C.
Zinc vitriol	ZnSO_4_•7H_2_O	99.5%	Xilong Chemical Industry Incorporated Co., Ltd., Guangdong Province, P.R.C.
Copper sulfate pentahydrate	CuSO_4_•5H_2_O	99.0%	Bodi Chemical Industry Incorporated Co., Ltd., Tianjin City, P.R.C.
Dichlorofluoroethane (R141b)	CH_3_-CCl_2_F	99.8%	Juhua Group Corporation, Zhejiang Province, P.R.C.
Ethylene glycol	(CH_2_OH)_2_	96.0%	Zhiao Chemical Reagent Institute, Liaoning Province, P.R.C.

**Table 2:** Specifications and suppliers of instruments.

Instrument	Model	Key Parameter	Supplier
Inductively coupled plasma optical spectrometer	Optima 2000DV	Detection limits: 1-10 ug/L, RSD ≤ 0.5%	PerkinElmer, United States
Raman spectroscopy	DXR	Laser wavelength: 532 nm	Thermo Fisher Scientific Co., Ltd., United States
ISCO pump	260D	Flow Range: 0.001-107 ml/min	Isco, Inc., United States
Data acquisition instrument	34972A	-	Agilent Co., United States
Low-temperature circulator	FP51	Precision: 0.01 °C	Julabo Co., Germany
Vacuum pump	SHB-111	Final vacuum: 0.098 MPa	Zhengzhou Greatwall Scientific Industrial and Trade Co, Ltd., P.R.C.
Centrifuge	TDZ5-WS	Max RPM: 5000 r/min	Xiangyi centrifuge instrument Co., Ltd., Hunan Province, P.R.C.

**Table 3:** Experimental conditions and results.

Sample	R141b–effluent volume ratio	Heavy metal ions	C_0_(mg/L)	C_11_(mg/L)	C_12_(mg/L)	C_2_(mg/L)	Re without washing (%)	Re with washing (%)	Ef	Yw (%)
S1	1:4	Cr^3+^	140.4	–	0.2108	257.5	–	99.85	1.8340	–
		Cu^2+^	143.9	–	0.3568	261.6	–	99.75	1.8179	
		Ni^2+^	136.5	–	0.2444	250.0	–	99.82	1.8315	
		Zn^2+^	133.7	–	0.3197	250.6	–	99.76	1.8743	
S2	1:6	Cr^3+^	96.70	28.99	9.936	126.4	70.02	89.72	1.3071	61.67
		Cu^2+^	104.4	29.37	9.583	133.9	71.87	90.82	1.2826	
		Ni^2+^	97.12	27.40	9.781	123.5	71.79	89.93	1.2716	
		Zn^2+^	93.36	30.04	11.19	122.6	67.82	88.01	1.3132	
S3	1:5	Cr^3+^	96.70	38.11	15.87	159.4	60.59	83.58	1.6487	71.67
		Cu^2+^	104.4	38.62	14.69	162.8	63.00	85.93	1.5599	
		Ni^2+^	97.12	37.06	15.26	155.6	61.83	84.28	1.6022	
		Zn^2+^	93.36	39.75	16.54	153.5	57.41	82.28	1.6442	
S4	1:4	Cr^3+^	96.70	45.45	23.12	178.9	53.00	76.09	1.8501	80.00
		Cu^2+^	104.4	45.54	22.56	190.5	56.38	78.39	1.8247	
		Ni^2+^	97.12	43.45	20.60	175.3	55.26	78.79	1.8050	
		Zn^2+^	93.36	46.63	22.39	174.5	50.05	73.13	1.8691	
